# Sex differences in autism spectrum disorder: behavioral and sensory phenotypes in humans and mouse models

**DOI:** 10.1038/s41398-026-04053-y

**Published:** 2026-05-01

**Authors:** Yuki Noriyama, Rio Ishida, Kazuhiko Yamamuro, Natsuko Kashida, Kazuki Okumura, Mamiko Okuda, Minobu Ikehara, Michihiro Toritsuka, Yasuhiko Saito, Takashi Okada, Nakao Iwata, Manabu Makinodan

**Affiliations:** 1 Department of Psychiatry, Nara Medical University, Kashihara, Nara 634-8522 Japan; 2 Department of Psychiatry, Fujita Health University, Toyoake, Aichi 470-1192 Japan; 3 Division of Transformative Psychiatry and Synergistic Research, International Centre for Brain Science, Fujita Health University, Toyoake, Aichi 470-1192 Japan; 4 Center for Health Control, Nara Medical University, Kashihara, Nara 634-8522 Japan; 5 Department of Neurophysiology, Nara Medical University, Kashihara, Nara 634-8521 Japan

**Keywords:** Neuroscience, Psychiatric disorders

## Abstract

Sex differences in autism spectrum disorder (ASD) are increasingly recognized, not only in symptom presentation but also in underlying neurobiology and response to environmental factors. However, current diagnostic practices and animal models are male-centric, overlooking female-specific phenotypes and mechanisms. We conducted a multimodal, cross-species study to assess sex-dependent ASD phenotypes. In high-functioning adults with ASD and typically developing (TD) controls, we evaluated self-reported autistic traits, self-reported sensory sensitivity, and clinician-observed behaviors using standardized tools: Autism-Spectrum Quotient, Adolescent/Adult Sensory Profile, and Autism Diagnostic Observation Schedule, Second Edition (ADOS-2). In parallel, we assessed behavioral phenotypes in a paternal 15q11–q13 duplication mouse model (15q dup/+) using open-field, light–dark transition, and augmented reality-based behavioral assays. Among humans, individuals with ASD showed greater self-reported sensory sensitivity and autistic traits than TD individuals. Within the ASD group, female participants reported greater self-reported sensory sensitivity and exhibited lower clinician-rated impairments (ADOS-2) than male participants, despite comparable self-reported autistic traits. No sex differences were found among TD individuals. In contrast, female 15q dup/+ mice exhibited heightened light-related sensory reactivity and reduced exploratory behavior under bright light. These findings suggest that sex differences in light-related sensory reactivity may be more readily detected through behavioral measures in animal models. Our findings underscore the importance of considering sex as a biological and behavioral variable in ASD research. Cross-species, phenotype-oriented approaches that integrate human and animal data may uncover subtle phenotypic variations and enhance sex-informed diagnostics and interventions.

## Introduction

Autism spectrum disorder (ASD) is a neurodevelopmental condition characterized by persistent impairments in social interaction and communication, along with restricted, repetitive behaviors and atypical sensory responses [1]. ASD was considered more prevalent and prototypical among male individuals. Although epidemiological data report a male-to-female prevalence ratio of approximately 3–4:1 [2, 3], this disparity is now attributed to diagnostic bias, including camouflaging behaviors in female individuals with ASD and male-centric conceptualizations of autistic traits [4, 5]. Reflecting these concerns, studies have begun to emphasize that ASD behavioral presentations, diagnosis, and neurobiology may differ by biological sex and socially constructed sex roles.

Growing evidence suggests that autistic traits may diverge in severity and qualitatively between sexes. Female individuals with ASD often exhibit more internalizing symptoms and fewer outwardly observable social difficulties [6, 7]. Some autistic females have socially normative interests (e.g., animals or literature), and less obvious stereotyped behaviors, hindering clinical recognition [8, 9]. Consequently, ASD among female individuals, particularly those without intellectual disability, is often diagnosed later in life [10], and is frequently misidentified as anxiety, depression, or a personality disorder [11].

Sex differences in self-reported sensory sensitivity offer additional insight into these diagnostic challenges. Over 85% of individuals with ASD experience atypical sensory responses [12], while female individuals with ASD report heightened sensitivity and greater emotional reactivity to sensory stimuli [13–15]. Studies using the Adolescent–Adult Sensory Profile (AASP) or the Sensory Processing Measure have shown that autistic females report greater auditory and balance/movement sensory sensitivity than autistic males [16, 17]. However, findings from meta-analyses remain mixed, with some studies reporting no significant sex differences across sensory domains [18].

From a neurobiological perspective, structural and functional imaging studies have documented sex-dependent patterns in ASD. Female individuals with ASD typically exhibit “neural masculinization”—e.g., increased left-lateralization in the auditory cortex, which contradicts the rightward asymmetry common among typically developing (TD) female individuals [19, 20]. Functional magnetic resonance imaging analyses have identified divergent connectivity patterns in motor, language, and visual networks between male and female individuals with ASD [21]. Moreover, female individuals with ASD rely more on striatal regions than the posterior superior temporal sulcus during social cognition, which may reflect neural adaptations associated with camouflaging strategies [22]. These patterns align with genomic findings, including a greater burden of rare deleterious mutations or copy number variants among female individuals with ASD, thus supporting the “female protective effect” hypothesis [23, 24].

Despite these advances, diagnostic practices and preclinical models remain largely male-centric. Standard assessments such as the Autism Diagnostic Observation Schedule, Second Edition (ADOS-2) were validated primarily on male individuals with ASD, resulting in male-biased samples [25, 26]. Similarly, preclinical rodent models of ASD have predominantly used male animals to avoid hormonal fluctuations linked to the estrous cycle, limiting the applicability to females [27]. However, studies using environmental and genetic mouse models—such as valproic-acid exposure or *Shank3/Cntnap2* deletions—have revealed both sex-dependent and sex-independent behavioral phenotypes [28, 29], underscoring the importance of incorporating sex as a biological variable. In human studies, “camouflaging” refers to strategies used to mask or compensate for autistic traits, primarily in social functioning, but may extend to other symptom domains [30].

In this study, we investigate sex-related differences in social behavior and self-reported sensory sensitivity among individuals with ASD and TD using both self-reported and clinician-rated assessments, including the Japanese version of the Autism-Spectrum Quotient (AQ-J), the ADOS-2, and the AASP. Further, to explore the cross-species relevance of these findings, we examined sex-dependent behavioral phenotypes using a well-established mouse model of ASD carrying a paternal duplication of the chromosomal region syntenic to human 15q11–q13 (15q dup/+) [31]. This study adopts a phenotype-oriented cross-species approach, avoiding assumptions of direct mechanistic equivalence between human ASD and mouse models, and cautiously explores shared behavioral dimensions. We hypothesize that ASD manifests in sex-dependent ways, shaped by both biological and sociocultural mechanisms in humans, and that sex-dependent behavioral patterns related to biological mechanisms can be explored across self-reported autistic traits, clinical evaluations, and preclinical behavioral data. Our findings aim to contribute to the understanding of sex-related heterogeneity in ASD and advance cross-species, phenotype-oriented approaches that integrate human and animal data in ASD research.

## Methods

### Human assessments

#### Participants

We recruited high-functioning individuals with ASD aged 18 years and older from patients who received outpatient or inpatient care at the Department of Psychiatry of Nara Medical University Hospital. Initial ASD diagnoses were conducted by at least two trained psychiatrists or psychologists based on the Diagnostic and Statistical Manual of Mental Disorders, Fifth Edition (DSM-5) [1]. We ensured diagnostic accuracy by only including individuals who fulfilled the ASD classification criteria based on the ADOS-2, Module 4 [32]. The ADOS-2 is the gold standard for objectively assessing social communication difficulties, restricted interests, and repetitive behaviors; studies have demonstrated its efficacy [33, 34]. Additionally, the Mini-International Neuropsychiatric Interview was administered to screen for psychiatric comorbidities [33]. Individuals with major psychiatric disorders or suicidal ideation were excluded. Detailed psychiatric comorbidities and medication status of the ASD sample are provided in Supplementary Tables Supplementary Table 1 and Supplementary Table 2, respectively. Participants with an estimated full-scale intelligence quotient < 70 were excluded to minimize the impact of intellectual disability on cognitive assessments, thus ensuring that the study focused on high-functioning individuals with ASD without co-occurring intellectual disabilities. The final sample included 253 individuals with ASD (178 male and 75 female individuals) who had an AQ-J score of ≥ 26 [35, 36]. We recruited TD controls through local print advertisements. TD participants underwent a standard clinical assessment comprising a psychiatric evaluation and a medical history examination conducted by two experienced psychiatrists. A psychologist evaluated the participants’ cognitive abilities utilizing the Wechsler Adult Intelligence Scale-Fourth Edition (WAIS). Finally, 152 TD individuals with no history or current diagnosis of ASD, psychiatric, or neurological disorders were included in the study. Participant recruitment, eligibility, enrollment, and inclusion in the final analyses are summarized in Supplementary Figure 1. The sample size was similar to that used in prior clinical studies examining sex differences in ASD using self-report measures and ADOS-2 assessments [4, 30]. ASD and TD groups were recruited to achieve comparable age and sex distributions at the group level; no significant differences were observed between groups (see Table 1).

**Table 1:** Participant demographics.

Variables	TD				ASD					
	Male		Female		Male		Female		*χ² or F* value	*p* value
	Mean	SD	Mean	SD	Mean	SD	Mean	SD		
Sex	N = 95		N = 57		N = 178		N = 75		2.667	0.125
Age	29.34	7.19	28.14	6.52	27.92	7.17	27.27	6.60	1.381	0.248
Estimated IQ	105.80	9.84	105.41	10.31	97.59	13.41	96.63	14.09	14.484	5.630 × 10^−9^

Age and estimated IQ are presented as means and standard deviations and were compared across groups using one-way ANOVA. Sex distribution (male/female) was compared between the ASD and TD groups using a chi-square test.

*TD* typically developing, *ASD* autism spectrum disorder, *SD* standard deviation, *IQ* intelligence quotient, *ANOVA* analysis of variance.

#### Assessment of autistic traits

Autistic traits were assessed using AQ-J, a self-reported questionnaire. The AQ-J was administered to both individuals with ASD and TD, thereby enabling direct comparisons across diagnostic categories. The ADOS-2 was administered only to individuals with ASD to confirm their diagnosis. Additionally, to allow for standardized assessment of autistic traits across both individuals with ASD and TD, we administered the self-reported AQ-J to all participants [37, 38].

#### Sensory sensitivity (self-reported)

Self-reported sensory sensitivity was assessed using the 60-item Adolescent–Adult Sensory Profile (AASP), a questionnaire evaluating individuals’ perceived responses to everyday sensory experiences [17, 39]. Items are rated on a five-point Likert scale and organized into four sensory response patterns: low registration, sensory seeking, sensory sensitivity, and sensation avoiding. The AASP is grounded in Dunn’s theoretical model of sensation processing, which conceptualizes sensory responses based on neurological thresholds and behavioral responses [40]. Higher scores on each scale reflect more pronounced atypical self-reported sensory responses in the corresponding domain.

### Animal behavioral procedures

The experimental design involved 15q dup/+ mice—a validated mouse model of ASD that carries a paternal duplication of the genomic region on mouse chromosome 7 C, orthologous to human 15q11–q13. This duplication model recapitulates core features of ASD, including social deficits and anxiety-like behaviors [31]. The 15q dup/+ mice were originally generated using chromosome engineering techniques on a C57BL/6 background to introduce a 6.3 Mb duplication spanning the Snrpn to Ube3a region, as first reported by Nakatani et al. [31], and have since been backcrossed to a C57BL/6 J background for more than 10 generations. Group sizes followed prior behavioral studies using the 15q dup/+ mouse model and are consistent with sample sizes commonly used in similar preclinical investigations [31, 41, 42]. The estrous cycle of female mice was not monitored. Female animals were tested without cycle staging and were randomly assigned across experimental conditions, in line with standard practice for group-level behavioral analyses. The mice were housed in a temperature- and humidity-controlled animal housing facility with a standard 12-hour light–dark cycle (8:00–20:00 lights on). All mice had free access to food and water. Experimental mice were all 15q dup/+, with age- and sex-matched (9–13 weeks old at the start of the behavioral experiment) wild-type (15q dup-) littermates as controls. Twenty-two mice of each genotype (15q dup/+ and 15q dup-) were used for general health checks, light–dark transition tests, open-field tests, and the augmented reality-based long-term animal behavior observing system (AR-LABO). All behavioral experiments were conducted during the light period (9 am–6 pm). All animal experiments were approved by the Nara Medical University Animal Care Committee and conducted in accordance with institutional guidelines. Procedures were consistent with the principles outlined in the National Institutes of Health Guide for the Care and Use of Laboratory Animals (8th ed.).

#### Augmented reality-based long-term animal behavior observing system

We applied our assay system for precise tracking and to analyze the social behavior of animals in a novel environment. This system determines the position of each mouse under social housing conditions. At least one week before the behavioral testing, each mouse was tagged with an identification (ID) marker (printed with ArUco Markers) on the back, attached via an elastic string under anesthesia (chloral hydrate 400 mg/kg, intraperitoneal administration). To prevent cage-mates from biting the ID tags, they were coated with quinine. In the behavioral experiment, one subject mouse and three age- and sex-matched C57BL/6 J mice that had never been co-housed with the subject mouse (i.e., unfamiliar mice) were placed in a cage (276 × 445 × 204 mm; CL-0128; CLEA Japan Inc., Tokyo, Japan) and allowed to interact freely for 1 h. The behavior of all four mice was monitored at 20 frames/s using an infrared camera under infrared illumination. The video data were analyzed offline using an in-house tracking syste [42, 43] (now available at O’Hara & Co., Ltd.). The central position of each mouse ID tag was detected in each frame as XY coordinates within the cage. The time-series coordinate data were exported as comma-separated values (.csv) files. The subsequent data processing was carried out using the same algorithm as we described previously [42, 43], using Python 3.10.8 and in-house programs. Missing coordinates were linearly interpolated between detected frames.

Locomotor activity was quantified as the total distance moved (in meters) by the ID tag of each mouse within a specified period. To analyze social interactions, we defined a contact event when the ID tags of two mice were within 20 mm of each other. Because a single distance cut-off can be arbitrary, we additionally repeated the contact-based analyses using two alternative proximity thresholds (25 and 30 mm) as a sensitivity analysis. For each threshold, a contact event was defined as a continuous period during which the distance between two mice remained below the selected threshold (i.e., transitions from <30 to <20 mm were not counted as separate contacts within the same threshold). Contact initiation was classified based on the distances moved by each mouse in the previous second (20 frames): the mouse moving the longer distance was labelled as the approaching mouse and the other mouse (moving the shorter distance) as the receiving mouse. Contact initiated by the approaching mouse was categorized as active contact, whereas contact experienced by the receiving mouse was categorized as passive contact. Subsequently, contact frequency and duration were calculated for each mouse in both active and passive roles. Additionally, to assess whether a mouse was biased toward a specific cage-mate, an entropy measure (Hi) was calculated based on the probability (pij) of contacting different mice using the following formula: Hi = −Σj {pij log2 (pij)}, where Hi represents the entropy of social interactions for mouse I, pij is the probability that mouse i interacts with mouse j (either in terms of number of interactions or total interaction duration), and the summation ∑j runs over all potential interaction partners j (i.e., the three cage-mates of mouse i).

#### Open-field test

An open-field test was conducted to evaluate exploratory activity and anxiety-like behavior [44]. The apparatus comprised an acrylic arena (22 × 44 × 40 cm). Each mouse was tested in the same arena under two illumination conditions (15 lx and 100 lx) in separate sessions using the same individuals. For each session, mice were placed in the arena and allowed to explore freely for 6 min. Locomotor distance (m), time spent in the center zone (s), and the number of center entries were recorded and analyzed using ANY-maze software v.6.34. Illumination order was counterbalanced across mice, with an inter-session interval of at least 24 h.

#### Light–dark transition tests

Light–dark transition tests were performed as previously described [45, 46]. A dark–light transition test assessed approach–avoidance behavior in response to illumination. The apparatus consisted of two compartments of equal size, one brightly illuminated (100 lx) with white walls and one dimly lit (15 lx) with black walls, separated by a small opening. Mice were initially placed in the dark compartment and allowed to freely explore both compartments for 6 min. The time spent in the light compartment (s) and the number of transitions between compartments were recorded using ANY-maze software v.6.34. To account for individual differences in locomotor activity, time-based measures were normalized to the total test duration.

#### Ethics statement

All procedures involving human participants were approved by the Certified Review Board of Nara Medical University (approval ID: 1319). The study followed the Declaration of Helsinki. Written informed consent was obtained from all participants after a complete description of the study, and participants were informed of their right to withdraw at any time without penalty.

All animal experiments were approved by the Nara Medical University Animal Care and Use Committee and followed institutional guidelines and the National Institutes of Health Guide for the Care and Use of Laboratory Animals (8th edition). The study was designed and reported in compliance with the ARRIVE guidelines.

### Statistical analyses

Primary outcomes were predefined as sex-related differences in sensory-related measures and social behavior, assessed using self-report questionnaires, clinician-rated assessments (ADOS-2), and corresponding behavioral indices in the mouse model. For analyses involving multiple comparisons within predefined outcome domains, appropriate corrections for multiple testing were applied as specified below. Analyses not specified as primary outcomes were exploratory and should be interpreted accordingly. Statistical analyses for human and mouse experiments were performed using IBM SPSS Statistics version 26 (IBM Corp.) and Prism version 9 (GraphPad Software Inc.) software, respectively. Sample sizes were determined based on prior studies with comparable designs. Detailed procedures for normality and variance testing, group comparisons, and corrections for multiple comparisons are described separately for human and mouse data below.

### Analysis of human data

Group differences were assessed using Student’s t-tests for normally distributed data with equal variances, Welch’s t-tests for data with unequal variances, or Mann–Whitney U tests for non-normally distributed data. For AQ-J and AASP scores, a two-way analysis of covariance (ANCOVA) examined the main effects and interactions of sex and diagnosis (ASD vs. TD), with estimated IQ as a covariate. Between-sex comparisons of ADOS-2 scores within individuals with ASD also used ANCOVA with IQ as a covariate. Bonferroni-corrected post-hoc tests were applied where appropriate, and false discovery rate correction (q < 0.05) was used to adjust for multiple comparisons. To examine the discrepancies between self-reported and clinician-observed autistic traits, we conducted a repeated-measures analysis of variance (RM-ANOVA) within the ASD group. Standardized Z-scores of AQ-J and ADOS-2 total scores were used as the within-subject factor “assessment method.” Estimated IQ was included as a between-subject covariate. We assessed the main effect of assessment method as well as its interactions with sex and IQ. Significant assessment method × sex and assessment method × IQ interactions were identified, indicating that the discrepancy between self-reported and clinician-rated traits varied by sex and cognitive ability. Estimated marginal means revealed that female individuals with ASD reported higher autistic traits on the AQ-J but were rated lower on the ADOS-2, whereas male individuals showed the opposite pattern. Data are presented as mean ± standard deviation (SD), and *p* < 0.05 was considered statistically significant.

### Analysis of mouse data

For behavioral outcomes in mice, including locomotor activity and center time in the open-field test, exploration metrics in the light–dark transition test, and social interaction measures in the AR-LABO system, normality was assessed using the Shapiro–Wilk test, and homogeneity of variance was evaluated using the F-test. Based on these assumptions, group comparisons (e.g., genotype and sex) were performed using either Student’s t-test or the Mann–Whitney U test. Right-skewed variables, including locomotor distance, number of transitions, contact frequency, and total contact duration, were log-transformed prior to testing. Two-way ANOVAs were used to assess the main effects and interactions of genotype (15q dup/+ vs. 15q dup-) and sex. For time-dependent outcomes in the light–dark transition test, three-way ANOVAs (genotype × sex × time) were conducted. Bonferroni-corrected post hoc comparisons were performed where appropriate. Repeated-measures ANOVAs were used for AR-LABO data. Spearman’s rank correlation coefficients were calculated to assess associations between genotype and behavioral measures. All data are reported as mean ± SEM, and *p* < 0.05 was considered statistically significant.

## Results

### Demographic characteristics

Group differences in age, estimated IQ, and sex distribution were examined across four groups: male individuals with ASD (n = 178), female individuals with ASD (n = 75), male TD individuals (n = 95), and female TD individuals (n = 57). No significant differences were observed in age (*p* = 0.248, *F* = 1.381) or sex (*p* = 0.125, *F* = 2.725) distribution across groups (Table 1). Estimated IQ was significantly lower among individuals with ASD compared with TD individuals (*p* = 5.630 × 10^−9^, *F* = 14.484) (Table 1). To facilitate interpretation of the cross-species design, a summary table mapping human measures to corresponding mouse behavioral tasks and their primary outcome domains is provided (Supplementary Table 3).

### Self-reported autistic traits and self-reported sensory sensitivity

**Fig. 1:**
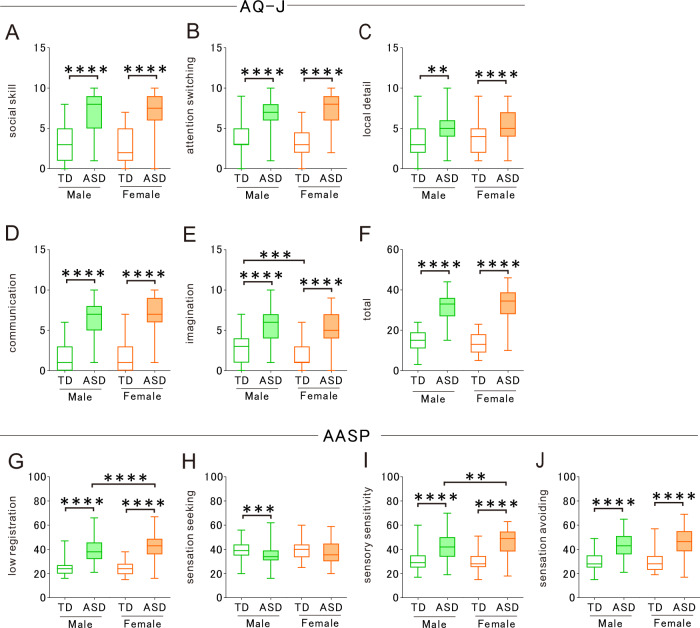
Diagnostic and sex-related differences in self-reported sensory sensitivity profiles assessed by the Adolescent/Adult Sensory Profile. **A–F** Mean AQ-J subscale scores for four groups: male individuals with ASD (n = 182), female individuals with ASD (n = 75), TD males (n = 95), and TD females (n = 56). Across all subscales—social skill (**A**), attention switching (**B**), local detail (**C**), communication (**D**), imagination (**E**) and total score (**F**),—scores were significantly higher in the ASD groups compared to the TD groups (post-hoc comparisons with Bonferroni-corrected **A**. TD vs. ASD in male individuals ****p = 2.990 × 10^-36^, TD vs. ASD in female individuals ****p = 5.845 × 10^-22^ **B**. TD vs. ASD in male individuals ****p = 8.393 × 10^-39^, TD vs. ASD in female individuals ****p = 2.035 × 10^−27^ **C**. TD vs. ASD in male individuals ****p = 9.235 × 10^−5^, TD vs ASD in female individuals **p = 0.001 **D**. TD vs. ASD in male individuals ****p = 9.289 × 10^−49^, TD vs. ASD in female individuals ****p = 1.273 × 10^−38^, **E**. TD vs. ASD in male individuals ****p = 3.073 × 10^−15^, TD vs. ASD in female individuals ****p = 4.341 × 10^−18^ **F**. TD vs. ASD in male individuals ****p = 5.358 × 10^−57^, TD vs. ASD in female individuals ****p = 2.538 × 10^−44^). For the imagination subscale (**E**), both a main effect of sex and a diagnosis × sex interaction were significant. Post-hoc comparisons revealed that female individuals with TD had significantly lower imagination scores than male individuals with TD (***p = 3.079 × 10^−4^, Bonferroni-corrected), while no significant difference was observed between ASD males and ASD females. **G–J** show the mean subscale scores on the AASP for four groups: male individuals with ASD (n = 182), female individuals with ASD (n = 75), male TD individuals (n = 95), and female TD individuals (n = 56). Across all subscales—low registration (**G**), sensation seeking (**H**), sensory sensitivity (**I**), and sensation avoiding (**J**)—scores were significantly higher in the ASD groups compared to the TD groups (Post-hoc comparisons with Bonferroni-corrected **G**. TD vs. ASD in male individuals ****p = 1.092 × 10^−28^, TD vs. ASD in female individuals ****p = 8.263 × 10^−28^ **H**. TD vs. ASD in male individuals ***p = 3.753 × 10^−4^, TD vs. ASD in female individuals *p* = 0.058 **I**. TD vs. ASD in male individuals ****p = 3.930 × 10^−17^, TD vs. ASD in female individuals ****p = 2.917 × 10^−17^ **J**. TD vs. ASD in male individuals****p = 4.392 × 10^−20^, TD vs. ASD in female individuals ****p = 1.468 × 10^−16^). For low registration (**G**) and sensory sensitivity (**I**), significant diagnosis × sex interactions were observed. Post-hoc comparisons indicated that female individuals with ASD scored higher than male individuals with ASD in both low registration (****p = 5.492 × 10^−5^, Bonferroni-corrected) and sensory sensitivity (**p = 0.001, Bonferroni-corrected). No significant sex differences were found within the TD group. Statistical analyses were conducted using two-way ANCOVA with estimated IQ as a covariate. Statistical significance was defined as **p < 0.01, ***p < 0.001, ****p < 0.0001. Abbreviation list: ASD = autism spectrum disorder; TD = typically developing; AQ-J = Autism-Spectrum Quotient, Japanese version; AASP = Adolescent/Adult Sensory Profile; ANCOVA = Analysis of Covariance; IQ = intelligence quotient.

Comparative analyses of self-reported autistic traits were performed utilizing the AQ-J. Significant main effects of diagnosis were identified for total AQ-J scores (*F*_1,400_ = 549.153, *p* = 4.607 × 10^−77^), as well as across all AQ-J subscales—social skill (*F*_1,400_ = 259.294, *p* = 2.499 × 10^−45^), attention switching (*F*_1,400_ = 312.091, *p* = 4.823 × 10^−52^), local detail (*F*_1,400_ = 22.113, *p* = 3.545 × 10^−6^), communication (*F*_1,400_ = 455.134, *p* = 5.519 × 10^−68^), and imagination (*F*_1,400_ = 146.249, *p* = 6.572 × 10^−29^) (Fig. 1, Table 2). These findings confirm that individuals with ASD exhibit markedly elevated levels of autistic traits, compared with TD individuals, as reflected across all measured domains. No significant sex differences were observed for total AQ-J scores (*F*₁,₄₀₀ = 0.001, *p* = 0.969) or for most subscales, including social skill (*F*₁,₄₀₀ = 0.960, *p* = 0.328), attention switching (*F*₁,₄₀₀ = 0.058, *p* = 0.810), local detail (*F*₁,₄₀₀ = 0.028, *p* = 0.868), and communication (*F*₁,₄₀₀ = 1.148, *p* = 0.285). Similarly, no significant diagnosis × sex interactions were found for total scores (*F*₁,₄₀₀ = 3.471, *p* = 0.063) or for most subscales (social skill: *F*₁,₄₀₀ = 0.030, *p* = 0.862; attention switching: *F*₁,₄₀₀ = 0.974, *p* = 0.324; local detail: *F*₁,₄₀₀ = 0.119, *p* = 0.730; communication: *F*₁,₄₀₀ = 3.564, *p* = 0.060).

**Table 2:** AQ-J scores by group and sex.

	TD		ASD				
Measure	Male Mean (SD)	Female Mean (SD)	Male Mean (SD)	Female Mean (SD)	Sex (F, p, Partial η²)	Diagnosis (F, p, Partial η²)	Interaction (F, p, Partial η²)
Total	15.48 (4.76)	14.16 (5.34)	30.99 (6.89)	32.23 (7.53)	*F*_1,400_ = 0.001 *p* = 0.969 Partial η² = 3.743 × 10^−6^	F_1,400_ = 549.153 *p* = 4.607 × 10^−77^ Partial η² = 0.579	F_1,400_ = 3.471 *p* = 0.063 Partial η² = 0.009
Social skill	2.73 (2.37)	2.93 (2.23)	6.94 (2.56)	7.23 (2.39)	*F*_1,400_ = 0.960 *p* = 0.328 Partial η² = 2.395 × 10^−3^	F_1,400_ = 259.294 *p* = 2.499 × 10^−45^ Partial η² = 0.393	F_1,400_ = 0.030 *p* = 0.862 Partial η² = 7.615 × 10^−5^
Attention switching	3.64 (1.74)	3.49 (1.75)	7.04 (1.91)	7.28 (1.79)	*F*_1,400_ = 0.058 *p* = 0.810 Partial η² = 1.449 × 10^−4^	F_1,400_ = 312.091 *p* = 4.823 × 10^−52^ Partial η² = 0.438	F_1,400_ = 0.974 *p* = 0.324 Partial η² = 0.002
Local details	4.07 (2.01)	4.04 (2.10)	5.13 (2.00)	5.25 (2.25)	*F*_1,400_ = 0.028 *p* = 0.868 Partial η² = 6.887 × 10^−5^	F_1,400_ = 22.113 *p* = 3.545 × 10^−6^ Partial η² = 0.052	F_1,400_ = 0.119 *p* = 0.730 Partial η² = 2.981 × 10^−4^
Communication	1.87 (1.72)	1.68 (1.75)	6.56 (2.45)	7.24 (2.01)	*F*_1,400_ = 1.148 *p* = 0.285 Partial η² = 0.003	F_1,400_ = 455.134 *p* = 5.519 × 10^−68^ Partial η² = 0.532	F_1,400_ = 3.564 *p* = 0.060 Partial η² = 0.009
Imagination	3.17 (1.70)	2.02 (1.52)	5.30 (2.01)	5.20 (2.16)	*F*_1,400_ = 9.242 *p* = 0.003 Partial η² = 0.023	F_1,400_ = 146.249 *p* = 6.572 × 10^−29^ Partial η² = 0.268	F_1,400_ = 6.370 *p* = 0.012 Partial η² = 0.016

Scores on the AQ-J and its five subscales are presented for each group (TD/ASD × male/female). A two-way ANCOVA was conducted to examine the main effects of sex and diagnosis, as well as their interaction, with estimated IQ included as a covariate.

*TD* typically developing, *ASD* autism spectrum disorder, *AQ-J* Autism-Spectrum Quotient, Japanese version, *SD*, standard deviation, *ANCOVA* analysis of variance.

However, for the imagination subscale, a significant main effect of sex (*F*₁,₄₀₀ = 9.242, *p* = 0.003) and a significant diagnosis × sex interaction (*F*₁,₄₀₀ = 6.370, *p* = 0.012) were observed, with female individuals with TD showing lower imagination scores than male individuals with TD, whereas no clear sex difference was observed within the ASD group (Fig. 1E, Table 2).

Self-reported sensory sensitivity was assessed using the AASP. Group comparisons revealed that individuals with ASD showed higher scores than TD individuals across all subscales, including low registration (*F*_1,400_ = 259.652, *p* = 2.241 × 10^−45^), sensation seeking (*F*_1,400_ = 13.573, *p* = 2.610 × 10^−4^), sensory sensitivity (*F*_1,400_ = 146.250, *p* = 6.569 × 10^−29^), and sensation avoiding (*F*_1,400_ = 153.167, *p* = 5.217 × 10^−30^) (Fig. 1G–J, Table 3). Moreover, significant diagnosis × sex interactions were observed for low registration (*F*_1,400_ = 5.773, *p* = 0.017) and sensory sensitivity (*F*_1,400_ = 4.205, *p* = 0.041). Given the significant diagnosis × sex interactions observed for low registration and sensory sensitivity, inspection of group means indicated that female individuals with ASD exhibited greater sensory sensitivity than their male counterparts in these domains, whereas no significant sex differences were observed within the TD group. These findings indicate that both diagnostic status and sex contribute to individual differences in sensory profiles, with heightened sensitivity observed among female individuals with ASD.

**Table 3:** AASP scores by group and sex.

	TD		ASD				
Measure	Male Mean (SD)	Female Mean (SD)	Male Mean (SD)	Female Mean (SD)	Sex (F, p, Partial η²)	Diagnosis (F, p, Partial η²)	Interaction (F, p, Partial η²)
Low registration	24.86 (6.12)	25.18 (6.15)	38.76 (10.20)	43.57 (8.63)	F_1,400_ = 7.378 *p* = 0.007 Partial η² = 0.018	F_1,400_ = 259.652 *p* = 2.241 × 10^−45^ Partial η² = 0.394	F_1,400_ = 5.773 *p* = 0.017 Partial η² = 0.014
Sensation seeking	39.09 (6.88)	38.53 (7.59)	35.63 (7.97)	36.36 (8.22)	F_1,400_ = 0.003 *p* = 0.957 Partial η² = 7.380 × 10^−6^	F_1,400_ = 13.573 *p* = 2.610 × 10^−4^ Partial η² = 0.033	F_1,400_ = 0.573 *p* = 0.449 Partial η² = 0.001
Sensory sensitivity	30.55 (7.34)	30.67 (7.44)	41.93 (11.06)	46.45 (11.27)	F_1,400_ = 4.676 *p* = 0.031 Partial η² = 0.012	F_1,400_ = 146.250 *p* = 6.569 × 10^−29^ Partial η² = 0.268	F_1,400_ = 4.205 *p* = 0.041 Partial η² = 0.010
Sensation avoiding	30.95 (7.48)	30.77 (8.56)	43.62 (10.42)	46.19 (11.58)	F_1,400_ = 1.219 *p* = 0.270 Partial η² = 0.003	F_1,400_ = 153.167 *p* = 5.217 × 10^−30^ Partial η² = 0.277	F_1,400_ = 1.639 *p* = 0.201 Partial η² = 0.004

Scores on the AASP and its four subscales are presented for each group (TD/ASD × male/female). A two-way ANCOVA was conducted to assess the main effects of sex and diagnosis, as well as their interaction, with estimated IQ included as a covariate.

*TD* typically developing, *ASD*, autism spectrum disorder, *AASP* Adolescent/Adult Sensory Profile, *SD* standard deviation, *ANCOVA* analysis of covariance, *IQ* intelligence quotient.

### Clinician-rated autistic symptoms (ADOS-2)

**Fig. 2:**
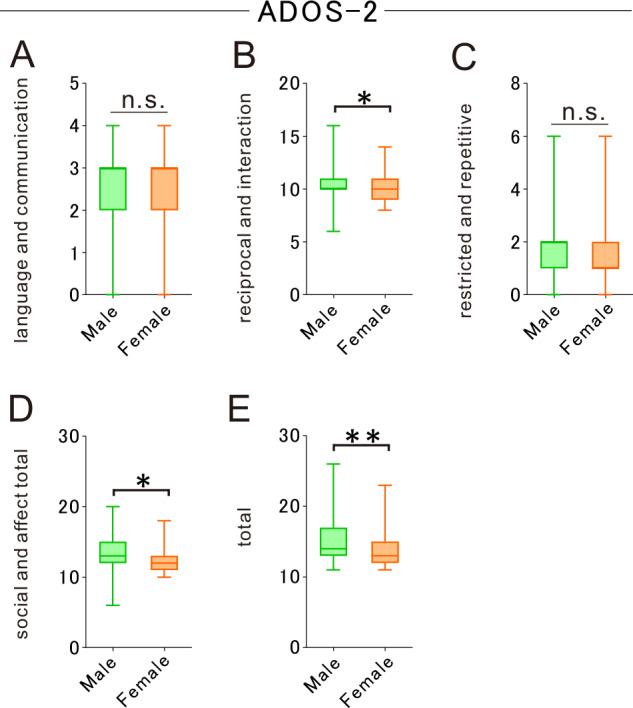
Clinician-rated autistic symptom severity based on ADOS-2 subscales. **A–E** Mean ADOS-2 subscale scores for male (n = 182) and female (n = 75) individuals with ASD. Compared to female individuals, male individuals exhibited significantly higher scores across all subscales: language and communication (**A**. *p = 0.036), reciprocal social interaction (**B**. *p = 0.019), restricted and repetitive behaviors (**C**. *p = 0.035), social affect total (**D**. **p = 0.008), and total score (**E**. **p = 0.003). Statistical comparisons were performed using an ANCOVA with estimated IQ as a covariate. All data are presented as box-and-whisker plots, where the central line indicates the median, the box represents the interquartile range (IQR; from the 25th percentile [Q1] to the 75th percentile [Q3]), and the whiskers extend to the minimum and maximum values excluding outliers. Statistical significance was defined as *p < 0.05, **p < 0.01. Abbreviation list: ADOS-2 = Autism Diagnostic Observation Schedule, Second Edition; ASD = autism spectrum disorder; ANCOVA = analysis of covariance; IQ = intelligence quotient; SEM = standard error of the mean.

To evaluate the severity of clinician-rated autistic symptoms, we compared ADOS-2 subscale scores between male (n = 182) and female (n = 75) individuals with ASD using an ANCOVA, controlling for estimated IQ. Male individuals exhibited significantly higher scores than female individuals across all subscales, including language and communication (*F*_1,250_ = 5.105, *p* = 0.025), reciprocal social interaction (*F*_1,250_ = 6.148, *p* = 0.014), restricted and repetitive behaviors (*F*_1,250_ = 4.646, *p* = 0.032), social affect (*F*_1,250_ = 7.957, *p* = 0.005), and the ADOS-2 total score (*F*_1,250_ = 9.586, *p* = 0.002) (Fig. 2, Table 4). These findings indicate that social and behavioral impairments are more prominently detected in male individuals during clinician-rated assessments. While self-reported measures revealed minimal sex differences, observational assessments captured clearer symptom expression in male individuals with ASD.

**Table 4:** ADOS-2 scores by sex in the ASD group.

	ASD		
Measure	Male Mean (SD)	Female Mean (SD)	F, p, Partial η²
Language and communication	2.91 (0.78)	2.56 (0.83)	F_1,250_ = 5.105 *p* = 0.025 Partial η² = 0.020
Reciprocal and interaction	10.62 (1.67)	10.09 (1.45)	F_1,250_ = 6.148 *p* = 0.014 Partial η² = 0.024
Restricted and repetitive stereotyped behaviors and interests	1.80 (1.06)	1.49 (1.16)	F_1,250_ = 4.646 *p* = 0.032 Partial η² = 0.018
Social affect	13.43 (2.14)	12.65 (1.80)	F_1,250_ = 7.957 *p* = 0.005 Partial η² = 0.031
Total	15.24 (2.67)	14.15 (2.53)	F_1,250_ = 9.586 *p* = 0.002 Partial η² = 0.037

ADOS-2 domain scores are presented separately for males and females within the ASD group. Between-sex comparisons were performed using ANCOVA with estimated IQ as a covariate.

*ASD* autism spectrum disorder, *ADOS-2* Autism Diagnostic Observation Schedule, Second Edition, *SD* standard deviation; *ANCOVA* analysis of covariance, *IQ* intelligence quotient.

### Discrepancies between self-reported and observed autistic traits: modulating effects of sex and IQ in individuals with ASD

A repeated-measures analysis of variance examined differences in autism-related traits assessed by self-report (AQ-J) and clinician observation (ADOS-2), after standardizing both measures into Z-scores based on the distribution within the ASD group. Estimated IQ was treated as a between-subject covariate (Table 5). There was a significant main effect of assessment method (*F*_1,250_ = 4.300, *p* = 0.039), indicating a discrepancy between standardized AQ-J and ADOS-2 scores. A significant interaction was observed between assessment method and estimated IQ (*F*_1,250_ = 5.174, *p* = 0.024), suggesting that the discrepancy between self-reported and observed autism traits varied as a function of IQ. Moreover, a significant interaction was found between assessment method and sex (*F*_1,250_ = 9.586, *p* = 0.002), indicating that male and female participants exhibited different patterns of discrepancy. Estimated marginal means revealed that female participants reported higher autistic traits on the AQ-J but were rated lower on the ADOS-2. In contrast, male participants reported lower traits on the AQ-J but were rated higher on the ADOS-2. These findings suggest that while the absolute severity of autistic traits may not differ by IQ or sex, both factors modulate the degree of discrepancy between self-perception and clinician evaluation. The observed pattern—particularly the tendency for females to report higher traits while presenting fewer observable symptoms—may reflect camouflaging behaviors, wherein individuals consciously or unconsciously mask their autistic characteristics in social contexts.

**Table 5:** Discrepancy between self-reported and observed autistic traits by sex in the ASD group.

	AQ-J		ADOS-2				
Measure	Male Mean (SD)	Female Mean (SD)	Male Mean (SD)	Female Mean (SD)	Assessment method (F, p, Partial η²)	Method×estimated IQ (F, p, Partial η²)	Method×sex (F, p, Partial η²)
Z-score	−0.06 (0.97)	0.11 (1.06)	0.12 (1.00)	−0.29 (0.95)	F_1,250_ = 4.300 *p* = 0.039 Partial η² = 0.017	F_1,250_ = 5.174 *p* = 0.024 Partial η² = 0.020	F_1,250_ = 9.586 *p* = 0.002 Partial η² = 0.037

Standardized scores (Z-scores) for self-reported autistic traits (AQ-J) and clinician-observed traits (ADOS-2) are plotted separately for male and female participants.

*ASD* autism spectrum disorder, *AQ-J* Autism-Spectrum Quotient, *ADOS-2* Autism Diagnostic Observation Schedule, Second Edition, *SD* standard deviation; *IQ* intelligence quotient.

### Light intensity-dependent modulation of exploratory behavior in 15q dup/+ mice

**Fig. 3:**
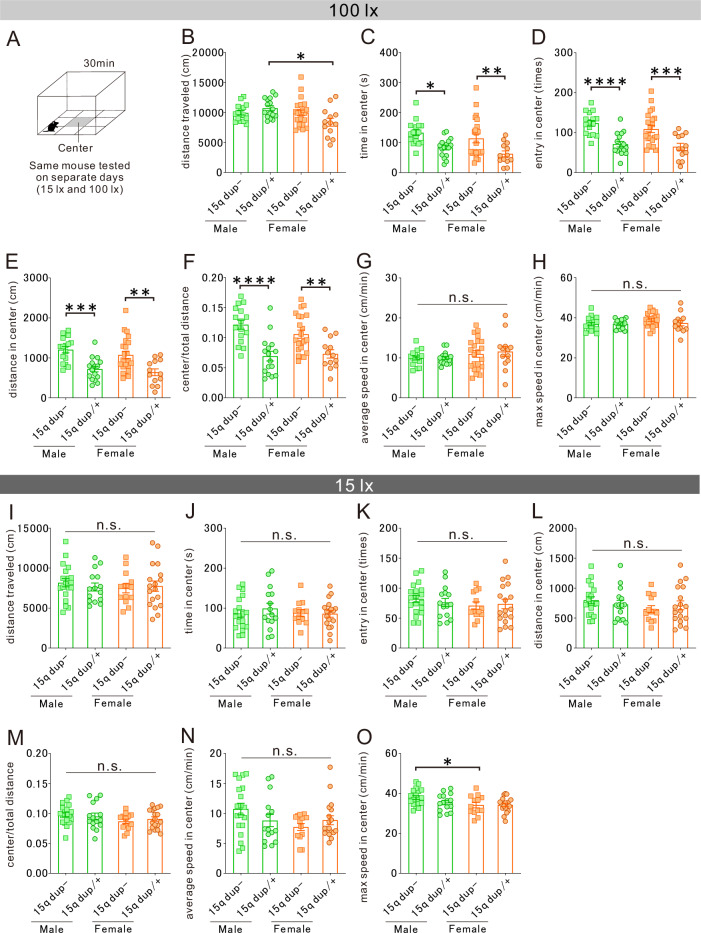
Light intensity-dependent differences in locomotion and anxiety-related behavior. **A** The same mice were tested under both 15 lux and 100 lux conditions on separate days. **B–H** Open field under the light condition (100 lx). **I–O** Open field under the dark condition (15 lx) (15q dup- male: 19 mice, 15q dup/+ male: 15 mice, 15q dup- female: 15 mice, 15q dup/+ female: 18 mice). **B** 15q dup/+ female mice exhibited reduced motor activity, compared with 15q dup/+ male mice (two-way ANOVA, genotype [15q dup- or 15q dup/+] × sex [male or female] interaction, *F*_1,63_ = 5.724, *p = 0.020; effect of genotype *F*_1,63_ = 0.686, *p* = 0.411; effect of sex *F*_1,63_ = 5.681, *p = 0.020, post-hoc tests with Bonferroni-corrected; 15q dup/+ male mice vs. 15q dup/+ female mice, *p = 0.014). **C–F** 15q dup/+ mice exhibited increased anxiety, compared with 15q dup- mice. (**C**. Two-way ANOVA, genotype [15q dup- or 15q dup/+] × sex [male or female] interaction, *F*_1,63_ = 0.004, *p* = 0.948; effect of genotype *F*_1,63_ = 17.270, ****p = 9.968 × 10^−5^; effect of sex *F*_1,63_ = 2.718, *p* = 0.104, post-hoc tests with Bonferroni-corrected; 15q dup/+ male vs. 15q dup- male mice, *p = 0.011, 15q dup/+ female vs. 15q dup- female mice, **p = 0.008; **D**. Two-way ANOVA, genotype [15q dup- or 15q dup/+] × sex [male or female] interaction, *F*_1,63_ = 0.219, *p* = 0.641; effect of genotype *F*_1,63_ = 33.370, ****p = 2.538 × 10^−7^; effect of sex *F*_1,63_ = 1.554, *p* = 0.217, post-hoc tests with Bonferroni-corrected; 15q dup/+ male vs. 15q dup- male mice, ****p = 8.753 × 10^−5^, 15q dup/+ female vs. 15q dup- female mice, ***p = 7.167 × 10^−4^; **E**. Two-way ANOVA, genotype [15q dup- or 15q dup/+] × sex [male or female] interaction, *F*_1,63_ = 0.103, *p* = 0.750; effect of genotype *F*_1,63_ = 26.720, ****p = 2.580 × 10^−6^; effect of sex *F*_1,63_ = 1.446, *p* = 0.234, post-hoc tests with Bonferroni-corrected; 15q dup/+ male vs. 15q dup- male mice, ***p = 5.365 × 10^−4^, 15q dup/+ female vs. 15q dup- female mice, **p = 0.002; **F**. Two-way ANOVA, genotype [15q dup- or 15q dup/+] × sex [male or female] interaction, *F*_1,63_ = 1.675, *p* = 0.200; effect of genotype *F*_1,63_ = 31.970, ****p = 4.080 × 10^−7^; effect of sex *F*_1,63_ = 0.624, *p* = 0.433, post-hoc tests with Bonferroni-corrected; 15q dup/+ male vs. 15q dup- male mice, ****p = 1.474 × 10^−5^, 15q dup/+ female vs. 15q dup- female mice, **p = 0.006) **G**. No differences in average speed in the center across genotype and sex (two-way ANOVA, genotype [15q dup- or 15q dup/+] × sex [male or female] interaction, *F*_1,63_ = 0.123, *p* = 0.727; effect of genotype *F*_1,63_ = 0.139, *p* = 0.710; effect of sex *F*_1,63_ = 2.623, *p* = 0.110). **H** No differences in max speed in the center across genotype and sex (two-way ANOVA, genotype [15q dup- or 15q dup/+] × sex [male or female] interaction, *F*_1,63_ = 0.938, *p* = 0.336; effect of genotype *F*_1,63_ = 0.980, *p* = 0.326; effect of sex *F*_1,63_ = 2.563, *p* = 0.114). **I** No differences in distance traveled across genotype and sex (two-way ANOVA, genotype [15q dup- or 15q dup/+] × sex [male or female] interaction, *F*_1,61_ = 0.491, *p* = 0.486; effect of genotype *F*_1,61_ = 0.055, *p* = 0.816; effect of sex *F*_1,61_ = 0.246, *p* = 0.621), compared with 15q dup- mice across sex. **J–M** 15q dup/+ mice exhibited no differences in anxiety (**J**. two-way ANOVA, genotype [15q dup- or 15q dup/+] × sex [male or female] interaction, *F*_1,61_ = 0.544, *p* = 0.464; effect of genotype *F*_1,61_ = 0.255, *p* = 0.616; effect of sex *F*_1,61_ = 0.316, *p* = 0.576; **K**. Two-way ANOVA, genotype [15q dup- or 15q dup/+] × sex [male or female] interaction, *F*_1,61_ = 0.400, *p* = 0.529; effect of genotype *F*_1,61_ = 0.055, *p* = 0.816; effect of sex *F*_1,61_ = 0.981, *p* = 0.326; **L**. Two-way ANOVA, genotype [15q dup- or 15q dup/+] × sex [male or female] interaction, *F*_1,61_ = 0.962, *p* = 0.331; effect of genotype *F*_1,61_ = 0.002, *p* = 0.963; effect of sex *F*_1,61_ = 1.440, *p* = 0.235; **M**. Two-way ANOVA, genotype [15q dup- or 15q dup/+] × sex [male or female] interaction, *F*_1,61_ = 0.695, *p* = 0.408; effect of genotype *F*_1,61_ = 0.008, *p* = 0.927; effect of sex *F*_1,61_ = 3.241, *p* = 0.077). **N**. No differences in average speed in the center across genotype and sex (two-way ANOVA, genotype [15q dup- or 15q dup/+] × sex [male or female] interaction, *F*_1,61_ = 2.962, *p* = 0.090; effect of genotype *F*_1,61_ = 0.196, *p* = 0.660; effect of sex *F*_1,61_ = 2.730, *p* = 0.104). **O**. 15q dup- male mice exhibited increased max speed in the center, compared with 15q dup- female mice (two-way ANOVA, genotype [15q dup- or 15q dup/+] × sex [male or female] interaction, *F*_1,61_ = 1.538, *p* = 0.220; effect of genotype *F*_1,61_ = 1.324, *p* = 0.254; effect of sex *F*_1,61_ = 5.604, *p = 0.021, post-hoc tests with Bonferroni-corrected; 15q dup- male vs. 15q dup- female mice, *p = 0.029). Abbreviation list: ANOVA = analysis of variance; 15q dup = 15q11–q13 duplication.

**Fig. 4:**
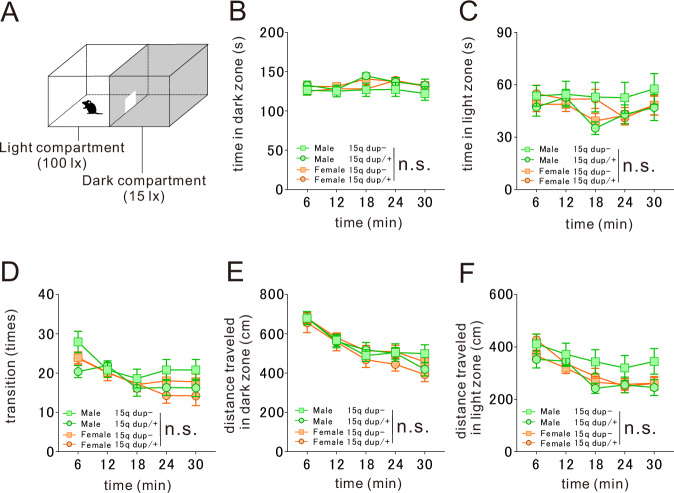
Locomotor and anxiety-related responses in the light–dark test. **A** The apparatus consisted of a light compartment (100 lux) and a dark compartment (15 lux). **B** There are no differences in time in the dark zone across genotype and sex (three-way ANOVA, genotype [15q dup- or 15q dup/+] × sex [male or female] × time [6, 12, 18, 24 or 30] interaction, *F*_4,236_ = 1.845, *p* = 0.121; genotype [15q dup- or 15q dup/+] × time [6, 12, 18, 24 or 30] interaction, *F*_4,236_ = 0.585, *p* = 0.674; sex [male or female] × time [6, 12, 18, 24 or 30] interaction, *F*_4,236_ = 0.868, *p* = 0.484; genotype [15q dup- or 15q dup/+] × sex [male or female] interaction, *F*_1,59_ = 1.706, *p* = 0.197). **C** No differences in time in the light zone across genotype and sex (three-way ANOVA, genotype [15q dup- or 15q dup/+) × sex [male or female] × time [6, 12, 18, 24 or 30] interaction, *F*_4,236_ = 1.845, *p* = 0.121; genotype [15q dup- or 15q dup/+] × time [6, 12, 18, 24 or 30] interaction, *F*_4,236_ = 0.585, *p* = 0.674; sex [male or female] × time [6, 12, 18, 24 or 30] interaction, *F*_4,236_ = 0.868, *p* = 0.484; genotype [15q dup- or 15q dup/+] × sex [male or female] interaction, *F*_1,59_ = 1.706, *p* = 0.197). **D** No differences in transition across genotype and sex (three-way ANOVA, genotype [15q dup- or 15q dup/+]× sex [male or female] × time [6, 12, 18, 24 or 30] interaction, *F*_4,236_ = 1.409, *p* = 0.232; genotype [15q dup- or 15q dup/+] × time [6, 12, 18, 24 or 30] interaction, *F*_4,236_ = 2.273, *p* = 0.062; sex [male or female] × time [6, 12, 18, 24 or 30] interaction, *F*_4,236_ = 0.634, *p* = 0.639; genotype [15q dup- or 15q dup/+] × sex [male or female] interaction, *F*_1,59_ = 0.396, *p* = 0.532). **E** No differences in distance traveled in the dark zone across genotype and sex (three-way ANOVA, genotype [15q dup- or 15q dup/+] × sex [male or female] × time [6, 12, 18, 24 or 30] interaction, *F*_4,236_ = 0.897, *p* = 0.466; genotype [15q dup- or 15q dup/+] × time [6, 12, 18, 24 or 30] interaction, *F*_4,236_ = 1.972, *p* = 0.100; sex [male or female] × time [6, 12, 18, 24 or 30] interaction, *F*_4,236_ = 0.646, *p* = 0.630; genotype [15q dup- or 15q dup/+] × sex [male or female] interaction, *F*_1,59_ = 0.249, *p* = 0.619). **F** No differences in distance traveled in the light zone across genotype and sex (three-way ANOVA, genotype [15q dup- or 15q dup/+] × sex [male or female] × time [6, 12, 18, 24 or 30] interaction, *F*_4,236_ = 0.855, *p* = 0.492; genotype [15q dup- or 15q dup/+] × time [6, 12, 18, 24 or 30] interaction, *F*_4,236_ = 1.295, *p* = 0.273; sex [male or female] × time [6, 12, 18, 24 or 30] interaction, *F*_4,236_ = 1.068, *p* = 0.373; genotype [15q dup- or 15q dup/+] × sex [male or female] interaction, *F*_1,59_ = 2.765, *p* = 0.102). Abbreviation list: ANOVA = analysis of variance; 15q dup = 15q11–q13 duplication.

In the open field test, exploratory behavior varied by lighting conditions (Fig. 3A). Given human findings of heightened sensory sensitivity, including light-related sensitivity, and sex differences in this domain, we investigated whether similar patterns emerged in 15q dup/+ mice. Female 15q dup/+ mice exhibited significantly reduced locomotion compared to male 15q dup/+ mice under bright light (100 lx) (Fig. 3B; genotype × sex interaction, *F*₁,₆₃ = 5.724, *p* = 0.020; main effect of sex, *F*₁,₆₃ = 5.681, *p* = 0.020). Under bright conditions, both male and female 15q dup/+ mice spent less time in the center, made fewer center entries, and showed reduced center distance compared to 15q dup− mice (Fig. 3C–E; main effect of genotype: center time, *F*₁,₆₃ = 17.270, *p* = 9.97 × 10⁻⁵; center entries, *F*₁,₆₃ = 33.370, *p* = 2.54 × 10⁻⁷; center distance, *F*₁,₆₃ = 26.720, *p* = 2.58 × 10⁻⁶), resulting in a decreased center-to-total distance ratio (Fig. 3F; main effect of genotype, *F*₁,₆₃ = 31.970, *p* = 4.08 × 10⁻⁷). No group differences were observed in average or maximum speed under bright light (Fig. 3G, H; all *p* > 0.3). In contrast, under low light conditions (15 lx), locomotion levels were comparable across genotypes and sexes (Fig. 3I; all *p* > 0.3), and no significant differences were observed in center-related measures, including time spent in the center, center entries, center distance, or center-to-total distance ratio (Fig. 3J–M; all *p* > 0.3). Under low light, average speed did not differ across groups, whereas maximum speed was higher in 15q dup− mice (Fig. 3N, O; main effect of sex, *F*₁,₆₁ = 5.604, *p* = 0.021). To further evaluate responses to brightness, we conducted a light–dark transition test (Fig. 4A). No significant group or sex differences were observed in time spent in the dark or light compartments, transitions, or exploration time (Fig. 4B–F; genotype × sex × time interaction for time in dark, *F*₄,₂₃₆ = 1.845, *p* = 0.121; genotype × sex interaction, *F*₁,₅₉ = 1.706, *p* = 0.197; all other *p* > 0.1). These results suggest that behavioral changes observed in the open field were specific to the bright-lit central zone, rather than reflecting generalized light preference or global sensory processing alterations.

### Sex- and genotype-dependent social approach in 15q dup/+ mice

**Fig. 5:**
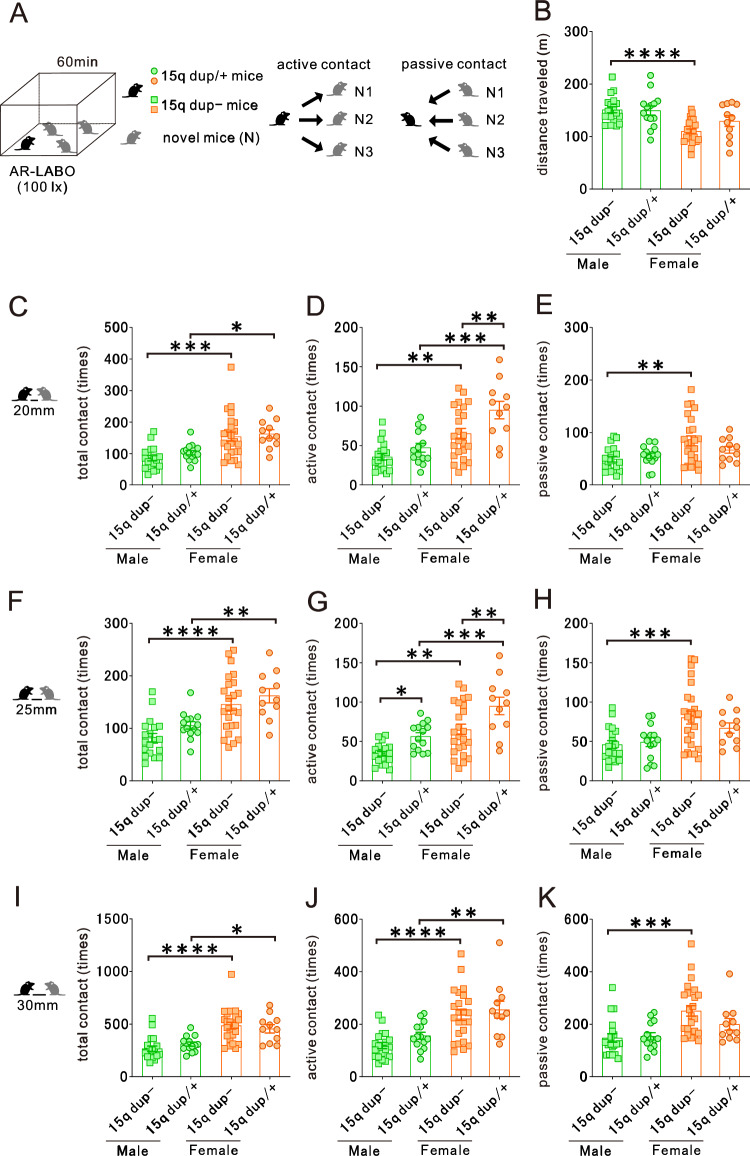
Sex-dependent social interaction in freely moving mice. **A** 15q dup- or 15q dup/+ mice freely interacted with three novel mice for 60 min (15q dup- male: 19 mice, 15q dup- female: 24 mice, 15q dup/+ male: 14 mice, 15q dup/+ female: 11 mice). **C-E** The social interaction threshold distance was defined as 20 mm. **F–H** The social interaction threshold distance was defined as 25 mm. **I–K** The social interaction threshold distance was defined as 30 mm. **B** 15q dup- male mice exhibited a higher traveled distance, compared with 15q dup- female mice (two-way ANOVA, genotype [15q dup- or 15q dup/+]× sex [male or female] interaction, *F*_1,64_ = 2.304, *p* = 0.134; effect of genotype *F*_1,64_ = 1.725, *p* = 0.194; effect of sex *F*_1,64_ = 19.650, ****p = 0.372 × 10^−4^, post-hoc tests with Bonferroni-corrected; 15q dup- male vs. 15q dup- female mice, *p = 0.133 × 10^−4^). **C** 15q dup + / male mice exhibited lower total contacts, compared with 15q dup- female mice (two-way ANOVA, genotype [15q dup- or 15q dup/+]× sex [male or female] interaction, *F*_1,64_ = 0.069, *p* = 0.794; effect of genotype *F*_1,64_ = 0.809, *p* = 0.372; effect of sex *F*_1,64_ = 22.160, ****p = 0.145 × 10^−4^, post-hoc tests with Bonferroni-corrected; 15q dup- male vs. 15q dup- female mice, ***p = 0.242 × 10^−3^, 15q dup/+ male vs. 15q dup/+ female mice, *p = 0.014). **D** 15q dup/+ female mice exhibited higher active contacts, compared with 15q dup- female mice (two-way ANOVA, genotype [15q dup- or 15q dup/+]× sex [male or female] interaction, *F*_1,64_ = 1.718, *p* = 0.195; effect of genotype *F*_1,64_ = 8.638, *p* = 0.005; effect of sex *F*_1,64_ = 29.810, ****p = 0.830 × 10^−6^, Post-hoc tests with Bonferroni-corrected; 15q dup- male vs. 15q dup- female mice, **p = 0.002, 15q dup/+ male vs. 15q dup/+ female mice, ***p = 0.238 × 10^−3^, 15q dup- female vs. 15q dup/+ female mice, **p = 0.009). **E** 15q dup- male mice exhibited lower passive contacts, compared with 15q dup- female mice (two-way ANOVA, genotype [15q dup- or 15q dup/+]× sex [male or female] interaction, *F*_1,64_ = 1.667, *p* = 0.201; effect of genotype *F*_1,64_ = 0.272, *p* = 0.604; effect of sex *F*_1,64_ = 8.764, **p = 0.004, post-hoc tests with Bonferroni-corrected; 15q dup- male vs. 15q dup- female mice, **p = 0.001). **F** 15q dup + / male mice exhibited lower total contacts, compared with 15q dup- female mice (two-way ANOVA, genotype [15q dup- or 15q dup/+]× sex [male or female] interaction, *F*_1,64_ = 0.006, *p* = 0.940; effect of genotype *F*_1,64_ = 2.432, *p* = 0.124; effect of sex *F*_1,64_ = 29.830, ****p = 0.822 × 10^−6^, post-hoc tests with Bonferroni-corrected; 15q dup- male vs. 15q dup- female mice, ***p = 0.461 × 10^−4^, 15q dup/+ male vs. 15q dup/+ female mice, **p = 0.002). **G** 15q dup/+ mice exhibited higher active contacts, compared with 15q dup- mice (two-way ANOVA, genotype [15q dup- or 15q dup/+]× sex [male or female] interaction, *F*_1,64_ = 0.350, *p* = 0.556; effect of genotype *F*_1,64_ = 14.800, ***p = 0.279 × 10^−3^; effect of sex *F*_1,64_ = 26.720, ****p = 0.251 × 10^−5^, post-hoc tests with Bonferroni-corrected; 15q dup- male vs. 15q dup- female mice, **p = 0.704 × 10^−3^, 15q dup/+ male vs. 15q dup/+ female mice, **p = 0.001, 15q dup- male vs. 15q dup/+ male mice, *p = 0.045, 15q dup- male vs. 15q dup/+ male mice, *p = 0.045, 15q dup- female vs. 15q dup/+ female mice, **p = 0.006). **H** 15q dup- male mice exhibited lower passive contacts, compared with 15q dup- female mice (two-way ANOVA, genotype [15q dup- or 15q dup/+]× sex [male or female] interaction, *F*_1,64_ = 1.219, *p* = 0.274; effect of genotype *F*_1,64_ = 0.455, *p* = 0.503; effect of sex *F*_1,64_ = 11.800, **p = 0.001, post-hoc tests with Bonferroni-corrected; 15q dup- male vs. 15q dup- female mice, ***p = 0.781 × 10^−4^). **I** 15q dup + / male mice exhibited lower total contacts, compared with 15q dup- female mice (two-way ANOVA, genotype [15q dup- or 15q dup/+]× sex [male or female] interaction, *F*_1,64_ = 1.321, *p* = 0.255; effect of genotype *F*_1,64_ = 0.042, *p* = 0.838; effect of sex *F*_1,64_ = 32.290, ****p = 0.351 × 10^−6^, post-hoc tests with Bonferroni-corrected; 15q dup- male vs. 15q dup- female mice, ***p = 0.843 × 10^−6^, 15q dup/+ male vs. 15q dup/+ female mice, *p = 0.012). **J** 15q dup + / male mice exhibited lower active contacts, compared with 15q dup- female mice (two-way ANOVA, genotype [15q dup- or 15q dup/+]× sex [male or female] interaction, *F*_1,64_ = 0.165, *p* = 0.686; effect of genotype *F*_1,64_ = 2.008, *p* = 0.161; effect of sex *F*_1,64_ = 27.850, ****p = 0.167 × 10^−5^, post-hoc tests with Bonferroni-corrected; 15q dup- male vs. 15q dup- female mice, ***p = 0.298 × 10^−4^, 15q dup/+ male vs. 15q dup/+ female mice, **p = 0.006). **K** 15q dup- male mice exhibited lower passive contacts, compared with 15q dup- female mice (two-way ANOVA, genotype [15q dup- or 15q dup/+]× sex [male or female] interaction, *F*_1,64_ = 2.322, *p* = 0.162; effect of genotype *F*_1,64_ = 1.405, *p* = 0.275; effect of sex *F*_1,64_ = 15.890, **p = 0.446 × 10^−3^, post-hoc tests with Bonferroni-corrected; 15q dup- male vs. 15q dup- female mice, ***p = 0.156 × 10^−3^). Abbreviation list: ANOVA = analysis of variance; 15q dup = 15q11–q13 duplication.

**Fig. 6:**
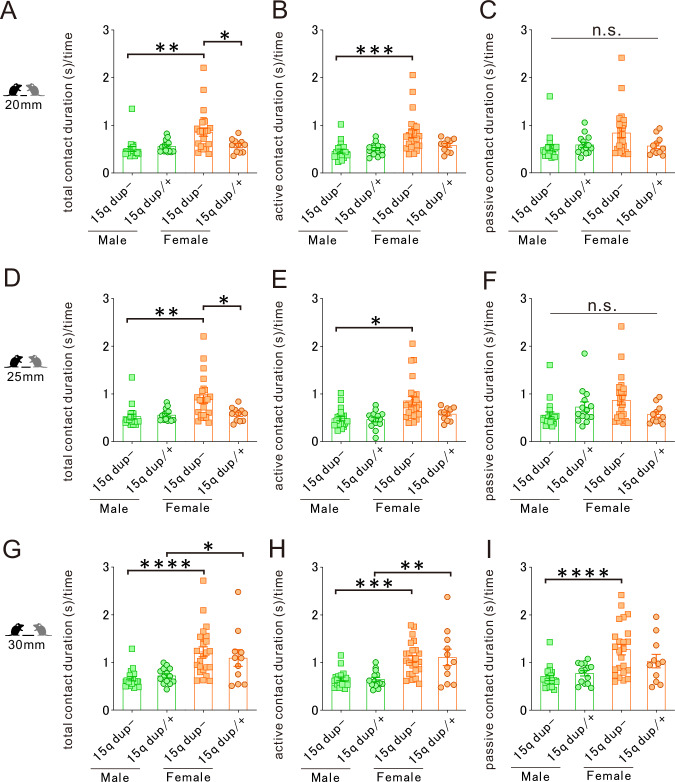
Quantification of social contacts and avoidance in freely moving mice. **A–C** The social interaction threshold distance was defined as 20 mm. **D–F** The social interaction threshold distance was defined as 25 mm. **G–I** The social interaction threshold distance was defined as 30 mm. **A** 15q dup/+ female mice exhibited lower total contact duration per time, compared with 15q dup- female mice (two-way ANOVA, genotype [15q dup- or 15q dup/+] × sex [male or female] interaction, *F*_1,64_ = 5.145, *p = 0.027; effect of genotype *F*_1,64_ = 2.422, *p* = 0.125; effect of sex *F*_1,64_ = 6.202, *p = 0.015, post-hoc tests with Bonferroni-corrected; 15q dup- male vs. 15q dup- female mice, ***p = 0.429 × 10^−3^, 15q dup- female vs. 15q dup/+ female mice, *p = 0.020). **B** 15q dup- male mice exhibited lower active contact duration per time, compared with 15q dup- female mice (two-way ANOVA, genotype [15q dup- or 15q dup/+] × sex [male or female] interaction, *F*_1,64_ = 5.006, *p = 0.041; effect of genotype *F*_1,64_ = 2.504, *p* = 0.145; effect of sex *F*_1,64_ = 11.860, **p = 0.002, post-hoc tests with Bonferroni-corrected; 15q dup- male vs. 15q dup- female mice, ***p = 0.917 × 10^−4^). **C** No differences in passive contact duration per time across genotype and sex (two-way ANOVA, genotype [15q dup- or 15q dup/+] × sex [male or female] interaction, *F*_1,64_ = 5.006, *p = 0.041; effect of genotype *F*_1,64_ = 2.504, *p* = 0.145; effect of sex *F*_1,64_ = 11.860, **p = 0.002). **D** 15q dup/+ female mice exhibited lower total contact duration per time, compared with 15q dup- female mice (two-way ANOVA, genotype [15q dup- or 15q dup/+] × sex [male or female] interaction, *F*_1,64_ = 5.038, *p = 0.028; effect of genotype *F*_1,64_ = 3.357, *p* = 0.072; effect of sex *F*_1,64_ = 6.039, *p = 0.017, post-hoc tests with Bonferroni-corrected; 15q dup- male vs. 15q dup- female mice, ***p = 0.501 × 10^−3^, 15q dup- female vs. 15q dup/+ female mice, *p = 0.012). **E** 15q dup- male mice exhibited lower active contact duration per time, compared with 15q dup- female mice (two-way ANOVA, genotype [15q dup- or 15q dup/+] × sex [male or female] interaction, *F*_1,64_ = 2.914, *p* = 0.093; effect of genotype *F*_1,64_ = 3.852, *p* = 0.054; effect of sex *F*_1,64_ = 10.370, **p = 0.002, post-hoc tests with Bonferroni-corrected; 15q dup- male vs. 15q dup- female mice, ***p = 0.269 × 10^−3^). **F** No differences in passive contact duration per time across genotype and sex (two-way ANOVA, genotype [15q dup- or 15q dup/+] × sex [male or female] interaction, *F*_1,64_ = 5.959, *p = 0.017; effect of genotype *F*_1,64_ = 0.444, *p* = 0.507; effect of sex *F*_1,64_ = 0.607, *p* = 0.439). **G** 15q dup + / male mice exhibited lower total contact duration per time, compared with 15q dup- female mice (two-way ANOVA, genotype [15q dup- or 15q dup/+] × sex [male or female] interaction, *F*_1,64_ = 0.889, *p* = 0.349; effect of genotype *F*_1,64_ = 0.111, *p* = 0.740; effect of sex *F*_1,64_ = 21.330, *p* = 0.192 × 10^−4^, post-hoc tests with Bonferroni-corrected; 15q dup- male vs. 15q dup- female mice, ***p = 0.429 × 10^−4^, 15q dup/+ male vs. 15q dup/+ female mice, *p = 0.048). **H** 15q ｄup + / male mice exhibited lower total contact duration per time, compared with 15q dup- female mice (two-way ANOVA, genotype [15q dup- or 15q dup/+] × sex [male or female] interaction, *F*_1,64_ = 0.080, *p* = 0.778; effect of genotype *F*_1,64_ = 0.015, *p* = 0.902; effect of sex *F*_1,64_ = 27.720, *p* = 0.175 × 10^−5^, post-hoc tests with Bonferroni-corrected; 15q dup- male vs. 15q dup- female mice, ***p = 0.230 × 10^−3^, 15q dup/+ male vs. 15q dup/+ female mice, **p = 0.002). **I** 15q dup- male mice exhibited lower passive contact duration per time, compared with 15q dup- female mice (two-way ANOVA, genotype [15q dup- or 15q dup/+] × sex [male or female] interaction, *F*_1,64_ = 2.862, *p* = 0.096; effect of genotype *F*_1,64_ = 0.475, *p* = 0.493; effect of sex *F*_1,64_ = 17.450, **p = 0.910 × 10^−4^, post-hoc tests with Bonferroni-corrected; 15q dup- male vs. 15q dup- female mice, ****p = 0.170 × 10^−4^). Abbreviation list: ANOVA = analysis of variance; 15q dup = 15q11–q13 duplication.

Social proximity and approach behaviors were assessed using the AR-LABO system under semi-naturalistic, mixed-sex housing conditions (Fig. 5A). Interactions were continuously tracked for 60 minutes under bright lighting (100 lx). Locomotor activity revealed a significant sex difference in 15q dup− mice, with males traveling farther than females (Fig. 5B; main effect of sex, *p* < 0.05); no such difference was observed in 15q dup/+ mice. Social approach behaviors were quantified at three distance thresholds (20, 25, and 30 mm). At the 20-mm threshold, female mice exhibited more total and active approaches than male mice (Fig. 5C, D; main effect of sex, all *p* < 0.05), and passive contacts were also higher in female 15q dup− mice compared with males (Fig. 5E; genotype × sex difference, *p* < 0.05). Similar patterns were observed at the 25-mm threshold, with females showing more total and active approaches (Fig. 5F, G; main effect of sex, all *p* < 0.05) and higher passive contacts in 15q dup− mice (Fig. 5H; *p* < 0.05). At the 30-mm threshold, females again exhibited more total and active approaches (Fig. 5I, J; main effect of sex, all *p* < 0.05), with higher passive contacts in female 15q dup− mice (Fig. 5K; *p* < 0.05). In addition, female 15q dup/+ mice showed more active approaches than female 15q dup− mice across thresholds. To evaluate the quality of social interactions, we calculated the average duration per approach. Female mice, especially 15q dup- females, showed longer contact durations than males at closer distances (20–25 mm), suggesting more persistent engagement, although this difference diminished at 30 mm (Fig. 6A–I; main effect of sex, all *p* < 0.01). In contrast, entropy analyses revealed no significant sex or genotype differences in contact diversity, indicating that female-biased approach behavior was not driven by preference for specific partners (sFig. 2A–I; all *p* > 0.1). These results suggest that female 15q dup/+ mice exhibit enhanced social approach and persistence in close-proximity interactions, which may reflect a sex-dependent compensatory or hyper-reactive behavioral phenotype. Such differences are not attributable to partner bias but reflect broader alterations in spontaneous social engagement.

## Discussion

This study identified sex differences in autistic traits across self-reported, observational, and sensory domains among individuals with ASD, along with corresponding behavioral phenotypes in a 15q dup/+ mouse model. These converging findings substantiate the relevance of sex as a biological and behavioral variable in ASD research.

In recent years, sex differences in ASD have been investigated in separate domains such as self-reported autistic traits [30], clinician-rated behaviors [47], and sensory sensitivity [13]. Furthermore, animal model studies reveal important sex differences in social behavior and stress responsivity [48]. However, few studies have integrated these multiple levels of assessment or cross-validated findings between human and animal models. Consequently, we systematically examined sex differences in self-perception, self-reported sensory sensitivity, and clinician-observed behaviors in a large sample of individuals with ASD and TD. Although sex-related differences have been reported in specific ADOS-2 domains, overall diagnostic capture appears comparable across sexes [49]. Additionally, we evaluated sex-related differences in social and sensory-related behaviors in a 15q dup/+ mouse model of ASD. These approaches allowed us to elucidate the multilayered nature of sex differences in ASD, as well as provide novel insights bridging human and animal research.

This study identified significant differences in self-reported autistic traits between individuals with ASD and typically developing individuals, based on scores from the AQ-J. Although male and female individuals with ASD showed slight differences in self-reported social difficulties, these did not reach statistical significance. This finding aligns with previous reports suggesting that female individuals with ASD may engage in camouflaging to navigate social situation [30], which could contribute to a discrepancy between internal experiences and externally observable behaviors [50]. A small pediatric study suggested that female individuals with ASD tend to score higher than male individuals on self-reported autistic traits [51], though such findings may not generalize to adults due to age-related developmental differences. Our findings from a large adult sample support the view that sex differences in self-reported social difficulties are minimal, thereby highlighting the need to reconsider diagnostic approaches for female individuals with ASD.

Significant group differences were observed in self-reported sensory sensitivity profiles between individuals with ASD and TD. Particularly, female individuals with ASD exhibited higher scores than male individuals on the low registration and sensory sensitivity subscales. These findings indicate heightened sensory sensitivity among female individuals with ASD, which may contribute to increased daily stress and greater challenges in social adaptation. Studies have extensively documented sensory over-responsivity among individuals with ASD [52], while heightened sensory sensitivity has been associated with elevated anxiety symptoms [53]. Furthermore, associations between self-reported sensory sensitivity atypicalities and anxiety symptoms have been reported in studies focusing on older children and adolescents [54], and sensory abnormalities have been proposed as a shared feature of both ASD and anxiety disorders [55]. Anxiety symptoms are highly prevalent among individuals with ASD, with evidence indicating that female individuals are at particularly elevated risk for internalizing problems such as anxiety and depression [56, 57]. Sensory hypersensitivity and anxiety tendencies may interact and synergistically exacerbate difficulties in social and emotional functioning. Additionally, in the 15q dup/+ mouse model, we observed illumination-dependent behavioral changes in the open field test. Under high illumination, exploratory behavior and center-zone activity were markedly suppressed, suggesting that hypersensitivity to environmental stimuli manifested as anxiety-like behavior. Similar sex differences in anxiety tendencies have been reported in other ASD mouse models; for example, in studies using the valproic acid model, female mice have exhibited greater anxiety-related behaviors, compared with males [56]. These convergent findings across human and animal models support the presence of sensory-emotional vulnerability among female individuals with ASD and suggest that sensory hypersensitivity may contribute to the development of sex differences in ASD phenotypes. Importantly, no significant genotype- or sex-related differences were observed in the light–dark transition test, which directly assesses light avoidance behavior. This finding indicates that the illumination-dependent changes observed in the open-field test are unlikely to reflect generalized aversion to light, but rather context-dependent modulation of exploratory activity under different environmental conditions. In interpreting these findings, it is important to note that the sensory measures used in the present study are based on self-report and therefore reflect self-reported sensory sensitivity rather than objectively measured sensory processing. Sex differences in self-reported sensory sensitivity may be influenced by factors such as male under-reporting or heightened vigilance and self-monitoring in females, rather than reflecting actual sensory differences.

Furthermore, we observed sex differences in observational assessments. Using the ADOS-2, male individuals with ASD exhibited significantly higher scores than female individuals on the dimensions of reciprocal social interaction, social affect total, and the ADOS-2 total score. These findings suggest that social difficulties may be more readily detected in male individuals with ASD through observational assessment**s**. Our results align with previous studies. For example, Rynkiewicz and Łucka reported that female individuals with ASD scored lower than male individuals in the verbal and gestural communication domains of the ADOS and ADOS-2, increasing the risk of misclassification as “non-spectrum” cases [51]. Similarly, Mandy et al. underscored that ADOS-2 may be better attuned to detecting male-typical behavioral presentations of ASD, whereas female presentations are generally overlooked [58]. The ADOS-2—developed and validated primarily using male samples—may be less sensitive to the subtler or atypical symptom presentations exhibited by some female individuals with ASD [4]. Another possible contributing factor is the impact of camouflaging behaviors. Hull et al. reported that camouflaging behaviors may be more common among female individuals with ASD than among male individuals [59], potentially obscuring observable autistic traits in structured assessment settings, such as the ADOS-2. However, this study did not directly assess such behaviors, and therefore, this interpretation should be considered speculative. Taken together, these findings indicate a systematic discrepancy between self-reported and clinician-rated autistic traits, particularly in females. The observed self-report–clinician discrepancy may reflect social camouflaging, although this interpretation is necessarily inferential. Future studies incorporating standardized measures of social camouflaging are needed to test this hypothesis more rigorously.

In sum, these findings suggest that the observed sex differences in ADOS-2 scores may reflect not only symptom differences but also potential measurement biases and the influence of camouflaging behaviors, though further investigation is needed to confirm these mechanisms. Our study highlights the importance of integrating self-reported measures, self-reported sensory sensitivity, and observational assessments to better capture sex-related heterogeneity in ASD.

In our analysis of social behavior using the 15q dup/+ mouse model, sex differences in spontaneous social approach behavior were observed. By applying the AR-LABO method under free-moving conditions, female mice exhibited significantly more active social approaches compared with male mice, whereas the presence of the 15q11–13 duplication mutation amplified this tendency. These findings may phenomenologically resemble certain characteristics observed among female individuals with ASD, such as heightened sensory responsivity and a greater propensity for camouflaging behaviors [58, 59]. Although increased social approach behaviors among mice might superficially suggest enhanced social motivation, they might reflect heightened reactivity to environmental stimuli rather than purely increased social interest. According to the social motivation hypothesis [60], ASD is associated with reduced sensitivity to social rewards. The increased social approach behavior observed among female 15q dup/+ mice may reflect sex differences in behavioral response patterns. However, extrapolating these findings to human ASD mechanisms, such as camouflaging behaviors, requires caution, given animal models’ inherent limitations. When considered alongside the illumination-dependent behavioral changes observed in the open-field test, these findings suggest that 15q dup/+ mice display heightened vulnerability to social and sensory environmental factors. Accordingly, the translational relevance of these findings should be understood as exploratory and phenotype-based, rather than as evidence of direct genetic or mechanistic correspondence across species. Our cross-species findings underscore the importance of considering sex differences when investigating ASD pathophysiology. Furthermore, they highlight the need for future clinical evaluations and interventions to transcend simple measures of symptom severity, emphasizing the nuanced characterization of behavioral styles and environmental responsiveness.

This study provides novel insights into sex differences in ASD by integrating multiple dimensions, including self-reported autistic traits (AQ-J), sensory responsivity (AASP), observational assessments (ADOS-2), and social behavior in a 15q dup/+ mouse model. While previous studies have typically focused on isolated aspects, our cross-species, multimodal approach significantly advances understanding of ASD’s heterogeneity. Clinically, our findings underscore the importance of considering sex-related factors in diagnosing, evaluating, and supporting individuals with ASD. Particularly, female individuals with ASD may exhibit heightened sensory sensitivities and behavioral patterns that are less easily detected by standard observational assessments [4, 59]. Therefore, comprehensive, multi-dimensional assessments are crucial to achieving more accurate diagnoses and tailored interventions. Among our key findings, we observed that sex differences were minimal in self-reported autistic traits, but became more evident in sensory profiles and observational assessments. Furthermore, sex differences in spontaneous social approach behaviors were detected in the 15q dup/+ mouse model, suggesting that genetic background and environmental responsivity interact to shape behavioral phenotypes.

Nevertheless, several limitations of this study warrant consideration. First, the cross-sectional design precluded examination of developmental trajectories or causal inferences regarding sex differences, sensory features, or masking-related behaviors over time. Second, the reliance on self-reported measures may have introduced biases related to subjective perception and social desirability, and because social camouflaging was not directly assessed, its contribution to the observed self-report–clinician discrepancy cannot be directly tested. Third, caution is required when translating findings across species. The lack of genetic stratification in the human cohort, the use of a single genetic mouse model, and the inability of animal models to capture sociocultural influences limit direct mechanistic correspondence between mouse and human findings. Accordingly, interpretations involving sociocultural mechanisms are restricted to the human data. Fourth, the animal experiments did not include explicit cognitive assessments and focused on sensory reactivity and spontaneous social interaction. Social behavior in the AR-LABO paradigm was assessed only under a strong illumination condition, and sensory manipulation was limited to the visual modality. Therefore, the cross-species comparison should be interpreted as reflecting light-specific sensory sensitivity and social behavioral styles rather than cognitive or generalized sensory phenotypes. Fifth, the estrous cycle was not monitored in female mice, and potential cycle-dependent effects on behavior were not assessed.

Overall, our findings highlight the need for capturing the nuanced interplay between autistic trait expression, sensory characteristics, social behavior, and environmental responsivity, thus contributing to a more comprehensive and individualized understanding of ASD phenotypes. Future research should incorporate longitudinal designs to explore how sex differences evolve across individuals’ developmental stages, employing multi-level assessments that combine self-reports, observations, and physiological indices. Moreover, the development and validation of sex-dependent, individualized intervention programs should be prioritized.

## Supplementary information

Supplemental material

## Data Availability

The datasets generated and/or analyzed during the current study are not publicly available due to privacy and ethical restrictions but are available from the corresponding author on reasonable request.
