# Supplementary material for: Sex differences in autism spectrum disorder: behavioral and sensory phenotypes in humans and mouse models
Source: Transl Psychiatry. 2026 May 1;16:314. doi: 10.1038/s41398-026-04053-y (PMC13276041; doi:10.1038/s41398-026-04053-y)
Supplement: Supplementary file 1 — Supplemental material [file 41398_2026_4053_MOESM1_ESM.docx]

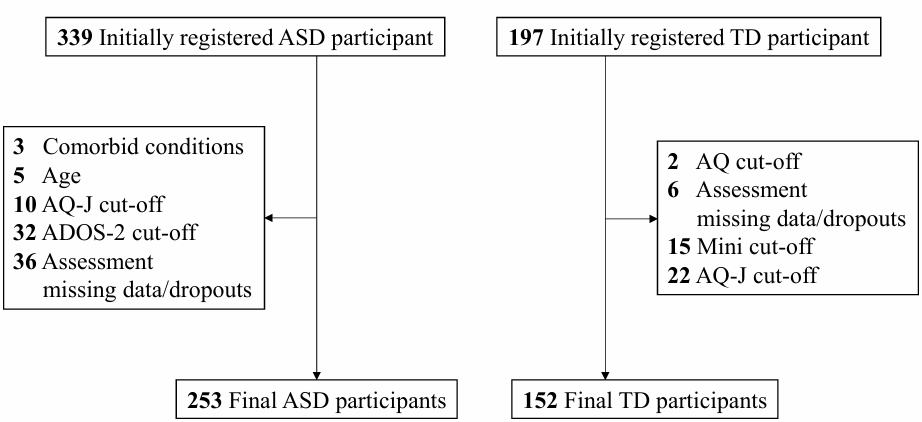


**Supplementary Figure 1. Participant flow diagram for ASD and TD groups**
This figure illustrates the participant selection process for the autism spectrum disorder (ASD) and typically developing (TD) groups. A total of 339 individuals were initially registered as ASD participants. After exclusions due to age (n = 5), comorbid conditions (n = 3), failure to meet the AQ-J cut-off (n = 10), failure to meet the ADOS-2 cut-off (n = 32), and missing assessment data or dropouts (n = 36), 253 ASD participants were included in the final analysis.

For the TD group, 197 participants were initially registered. Participants were excluded based on the AQ cut-off (n = 2), missing assessment data or dropouts (n = 6), failure to meet the Mini cut-off (n = 15), and failure to meet the AQ-J cut-off (n = 22). The final TD sample consisted of 152 participants.

**Abbreviations:** ASD, autism spectrum disorder; TD, typically developing; AQ, Autism-Spectrum Quotient; AQ-J, Japanese version of AQ; ADOS-2, Autism Diagnostic Observation Schedule–2; Mini, Mini-International Neuropsychiatric Interview.


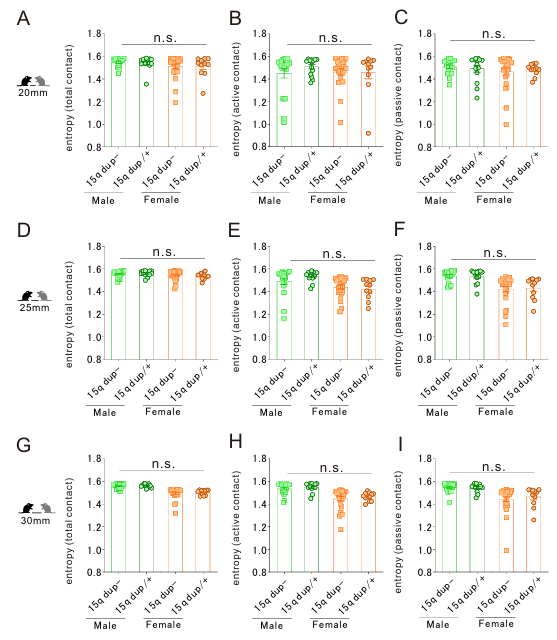


**Supplementary Figure 2. Assessment of social behavior in freely moving mice**

**A–C.** The social interaction threshold distance was defined as 20 mm. **D–F.** The social interaction threshold distance was defined as 25 mm. **G–I.** The social interaction threshold distance was defined as 30 mm. **A.** No differences in entropy of total contacts across genotype and sex (two-way ANOVA, genotype [15q dup- or 15q dup/+] × sex [male or female] interaction, *F*_1,64_ = 0.023, *p =* 0.880; effect of genotype *F*_1,64_ = 0.051, *p =* 0.821; effect of sex *F*_1,64_ = 2.943, *p =* 0.091). **B.** No differences in entropy of active contacts across genotype and sex (two-way ANOVA, genotype [15q dup- or 15q dup/+] × sex [male or female] interaction, *F*_1,64_ = 0.390, *p =* 0.535; effect of genotype *F*_1,64_ = 0.242, *p =* 0.624; effect of sex *F*_1,64_ = 0.539, *p =* 0.465). **C.** No differences in entropy of passive contacts across genotype and sex (two-way ANOVA, genotype [15q dup- or 15q dup/+] × sex [male or female] interaction, *F*_1,64_ = 0.278, *p =* 0.600; effect of genotype *F*_1,64_ = 0.958×10^-5^, *p =* 0.998; effect of sex *F*_1,64_ = 0.819, *p =* 0.369). **D.** No differences in entropy of total contacts across genotype and sex (two-way ANOVA, genotype [15q dup- or 15q dup/+] × sex [male or female] interaction, *F*_1,64_ = 0.713, *p =* 0.402; effect of genotype *F*_1,64_ = 0.017, *p =* 0.898; effect of sex *F*_1,64_ = 3.374, *p =* 0.071). **E.** No differences in entropy of active contacts across genotype and sex (two-way ANOVA, genotype [15q dup- or 15q dup/+] × sex [male or female] interaction, *F*_1,64_ = 0.097, *p =* 0.756; effect of genotype *F*_1,64_ = 0.251, *p =* 0.618; effect of sex *F*_1,64_ = 4.027, **p =* 0.049). **F.** No differences in entropy of passive contacts across genotype and sex (two-way ANOVA, genotype [15q dup- or 15q dup/+] × sex [male or female] interaction, *F*_1,64_ = 5.959, **p =* 0.017; effect of genotype *F*_1,64_ = 0.444, *p =* 0.507; effect of sex *F*_1,64_ = 0.607, *p =* 0.439). **G.** No differences in entropy of total contacts across genotype and sex (two-way ANOVA, genotype [15q dup- or 15q dup/+] × sex [male or female] interaction, *F*_1,64_ = 0.143, *p =* 0.707; effect of genotype *F*_1,64_ = 0.437, *p =* 0.511; effect of sex *F*_1,64_ = 0.338, *p =* 0.563). **H.** No differences in entropy of active contacts across genotype and sex (two-way ANOVA, genotype [15q dup- or 15q dup/+] × sex [male or female] interaction, *F*_1,64_ = 0.652, *p =* 0.422; effect of genotype *F*_1,64_ = 2.648, *p =* 0.109; effect of sex *F*_1,64_ = 0.026, *p =* 0.872). **I.** No differences in entropy of passive contacts across genotype and sex (two-way ANOVA, genotype [15q dup- or 15q dup/+] × sex [male or female] interaction, *F*_1,64_ = 0.275, *p =* 0.602; effect of genotype *F*_1,64_ = 0.119, *p =* 0.731; effect of sex *F*_1,64_ = 1.951, *p =* 0.167).

Abbreviation list: ANOVA = analysis of variance; 15q dup = 15q11–q13 duplication.

**Supplementary Table 1. Psychiatric comorbidities of the ASD sample (N = 253)**

| Comorbidities | N (%) |
| --- | --- |
| Attention-Deficit/Hyperactivity Disorder | 28 (11.1 %) |
| Major Depressive Disorder | 12 (4.7 %) |
| Obsessive-Compulsive Disorder | 4 (1.6 %) |
| Adjustment Disorders | 4 (1.6 %) |
| Dysthymia | 3 (1.2 %) |
| Alcohol Use Disorder | 2 (0.8 %) |
| Anxiety Disorders | 2 (0.8 %) |
| Borderline Personality Disorder | 1 (0.4 %) |
| Developmental Coordination Disorder | 1 (0.4 %) |
| Dissociative Disorders | 1 (0.4 %) |
| Eating Disorders | 1 (0.4 %) |
| Learning Disorder | 1 (0.4 %) |
| Obsessive-Compulsive Personality Disorder | 1 (0.4 %) |
| Somatic Symptom and Related Disorders | 1 (0.4 %) |

Values are presented as n (%). Percentages were calculated based on the total autism spectrum disorder (ASD) sample (N = 253). Psychiatric diagnoses were based on DSM-5 criteria. The total number represents the sum of comorbid conditions; overall, 52 participants had at least one comorbid diagnosis, and some participants had more than one comorbid diagnosis.

**Supplementary Table 2. Medication categories in the ASD sample (N = 253)**

| Medication categories | N (%) |
| --- | --- |
| Antidepressants | 82 (32.4%) |
| Benzodiazepines | 73 (28.9%) |
| Atypical antipsychotics | 50 (19.8%) |
| Mood stabilizers / antiepileptic drugs | 22 (8.7%) |
| ADHD medications | 21 (8.3%) |
| Non-benzodiazepine hypnotics | 20 (7.9%) |
| Non-benzodiazepine anxiolytics | 9 (3.6%) |
| Orexin receptor antagonists | 7 (2.8%) |
| Typical antipsychotics | 5 (2.0%) |
| Antiparkinsonian drugs | 3 (1.2%) |

Values are presented as n (%). Percentages were calculated based on the total autism spectrum disorder (ASD) sample (N = 253). Multiple medications per participant were allowed.

Abbreviations: ADHD, Attention-Deficit/Hyperactivity Disorder.

**Supplementary Table 3. Cross-species mapping of human measures and mouse behavioral tasks**

| **Symptom / Domain** | **Human Measure** | **Primary Outcome** | **Mouse Task** | **Primary Behavioral Index** |
| --- | --- | --- | --- | --- |
| Sensory sensitivity/avoidance | AASP (Sensory Sensitivity, Sensation Avoiding) | Heightened sensory responsivity | Open field test (bright vs dim light conditions) | Center time, center distance ratio |
| Light-related sensory response | AASP (Sensory Sensitivity) | Sensitivity to sensory input | Light–dark transition test | Time in light/dark compartments |
| Social behavior (observational) | ADOS-2 (Social Affect) | Observable social interaction patterns | Social approach task (AR-LABO) | Frequency and duration of active social approaches |
| Autistic traits (self-report) | AQ-J | Subjective autistic traits | — | — |

This table summarizes the correspondence between human clinical and self-report measures and the behavioral tasks used in the 15q dup/+ mouse model. Human measures are paired with mouse tasks based on shared symptom domains and primary behavioral outcomes, with the aim of facilitating interpretation of the cross-species design. The mappings are phenotype-based and intended to aid conceptual alignment rather than to imply direct mechanistic equivalence across species.

Abbreviations: AASP, Adolescent/Adult Sensory Profile; ADOS-2, Autism Diagnostic Observation Schedule, Second Edition; AQ-J, Autism-Spectrum Quotient, Japanese version.
